# Foot placement coordination is impaired in people with Parkinson’s disease

**DOI:** 10.1186/s12984-025-01830-6

**Published:** 2025-12-06

**Authors:** Charlotte Lang, Jeffrey M. Hausdorff, Sjoerd M. Bruijn, Matthew A. Brodie, Yoshiro Okubo, Walter Maetzler, Moira van Leeuwen, Navrag B. Singh, Jaap H. van Dieen, Deepak K. Ravi

**Affiliations:** 1 Laboratory for Movement Biomechanics, Institute for Biomechanics, Department of Health Sciences & Technology, ETH Zürich, Gloriastrasse 37/39, Zürich, 8092 Switzerland; 2 Center for the Study of Movement, Cognition, and Mobility, Neurological Institute, Tel Aviv Medical Center, Tel Aviv, Israel; 3 Department of Physical Therapy, Faculty of Medical & Health Sciences, Tel Aviv University, Tel Aviv, Israel; 4 Department of Human Movement Science, Vrije Universiteit Amsterdam, Amsterdam Movement Sciences, Amsterdam, Netherlands; 5 Graduate School of Biomedical Engineering, University of New South Wales, Sydney, NSW Australia; 6 Falls, Balance and Injury Research Centre, Neuroscience Research Australia, Randwick, Australia; 7 School of Population Health, University of New South Wales, Sydney, Australia; 8 Department of Neurology, University Hospital Schleswig-Holstein and Kiel University, Kiel, Germany; 9 Singapore-ETH Centre, Future Health Technologies Program, CREATE campus, 1 CREATE Way, #06-01 CREATE Tower, Singapore, 138602 Singapore

**Keywords:** Foot placement, Motor control, Balance, Walking, Parkinson’s disease

## Abstract

**Background:**

Gait instability is a common and disabling symptom of Parkinson’s disease (PD), contributing to frequent falls and reduced quality of life. While clinical balance tests and spatiotemporal gait measures can predict fall risk, they do not fully explain the underlying control mechanisms. In healthy individuals, foot placement is actively adjusted based on an estimate of the Center of Mass (CoM) state to maintain gait stability, known as foot placement control. This estimation relies on the integration of multisensory information, which has been shown to be impaired in PD, potentially disrupting the control of gait stability through foot placement. This study aimed to investigate whether foot placement coordination during overground walking is impaired in people with PD.

**Methods:**

Fifty people with PD and 51 healthy older adults walked overground for 10 min at self-selected walking speed. Foot placement errors were quantified as the deviation between the actual foot placement and the predicted placement derived from the CoM kinematic state during the preceding swing phase.

**Results:**

Foot placement errors were significantly higher in people with PD than in healthy older adults in both mediolateral (*p* < 0.05) and anteroposterior directions (*p* < 0.0001), at both mid-swing and terminal swing. Relative explained variance in mediolateral direction was significantly higher in people with PD compared to healthy older adults (*p* < 0.005).

**Conclusion:**

We provide first evidence of impaired coordination between the CoM and foot placement in PD. Future work should investigate a causal relationship between impaired foot placement control, sensorimotor integration and gait instability.

**Supplementary Information:**

The online version contains supplementary material available at 10.1186/s12984-025-01830-6.

## Background

Gait instability is a common and disabling symptom of Parkinson’s disease (PD). People with PD often walk slowly with short, shuffling and unstable steps, which increases their risk of falling [1]. Up to 50% of individuals with PD experience at least one fall, contributing significantly to reduced quality of life [1–4]. To assess these mobility deficits, clinicians and researchers commonly rely on two complementary approaches. The first involves clinical balance tests, such as the BESTest and miniBESTest [5, 6], which evaluate postural control across different tasks and have strong predictive value in distinguishing fallers from non-fallers. Other commonly used assessments include the Functional Gait Assessment [7], which assesses dynamic balance during walking, and the Berg-Balance-Scale [8, 9], which focuses exclusively on static balance. While these tools are practical and widely used, they can exhibit ceiling effects and provide limited insight into the specific mechanisms underlying instability [10].

To complement these clinical assessments, laboratory-based gait analyses, which quantify walking characteristics such as speed, step length, and step width are frequently used. These measures have been shown to differ significantly between people with PD and healthy adults and are strongly associated with fall risk [11–15]. In particular, increased variability in these parameters reflects reduced stability control during walking and is among the most robust predictors of future falls in PD [16]. However, even these objective gait metrics do not fully reveal the specific control mechanisms that give rise to gait instability in PD. Identifying these mechanisms may help to explain why some individuals remain stable while others experience falls in PD and may guide the development of more targeted interventions.

Gait stability relies on the ability to maintain the body’s Center of Mass (CoM) withinin the limits of the base of support to maintain balance. In quiet standing, this can be achieved through adjustments of body posture or ankle torque [17, 18]. During walking, however, stability control becomes considerably more complex because the CoM moves dynamically above a narrow and continuously shifting base of support. In healthy individuals, steady-state walking is primarily stabilized through foot placement control [19, 20]. With this mechanism, the position of each step, defined by its length and width (i.e., step length and step width) is adjusted based on the estimated state of the CoM, specifically its position and velocity, during the preceding swing phase [19, 20]. This allows for deviations in CoM movement from a stable trajectory to be accommodated by actively stepping in the direction of the disturbance, thereby maintaining gait stability. For example, if the CoM shifts laterally toward one side due to a perturbation or imbalance, the subsequent step is placed wider in that direction to realign the body’s CoM over the base of support. Consequently, steps tend to be wider when the CoM moves further or faster away from the stance foot. Here, the degree of foot placement control can be quantified by how well foot placement is predicted from the CoM kinematic state during the swing phase of walking, that is, from its position and velocity relative to the stance foot, which together determine the direction and magnitude of the corrective step required to maintain stability [19]. The aim of this predictive approach is to quantify the strength of the feedback control relationship between CoM motion and subsequent step placement. A strong predictive relationship indicates that foot placement is actively regulated in response to CoM dynamics.

In healthy young adults, this relationship is strong, with CoM dynamics explaining more than 80% of the variance in mediolateral foot placement [19]. Moreover, during the swing phase, the CoM state predicts subsequent foot placement even more accurately than the kinematic state of the swing foot itself [19]. Together with findings that sensory perturbations alter this relationship [21, 22], these results support the interpretation that foot placement control is an actively regulated feedback control mechanism guided by CoM kinematics during the preceding swing phase [20]. This mechanism plays a critical role in maintaining dynamic balance during walking [20].

In contrast, the degree of foot placement control has been shown to be reduced in older adults, as well as in individuals with stroke and incomplete spinal cord injury [21, 23, 24], highlighting its susceptibility to age- and disease-related changes in sensorimotor function. In older adults, foot placement is less tightly coupled to CoM motion compared with young adults, whereas in people with stroke, particularly those at high risk of falling, the ability to adjust foot placement in response to CoM variations is impaired in the paretic limb [23]. Such diminished control may therefore represent a key mechanism underlying gait instability and contribute to an elevated risk of falls in these populations.

Accurate estimation of the CoM state and appropriate foot placement adjustments rely on the integration of sensory information from the vestibular, visual and proprioceptive systems [20, 25, 26]. In people with PD, impairments in sensory processing and sensorimotor integration have been previously reported [27–30], which may compromise CoM state estimation and reduce the precision of foot placement. While foot placement control has been studied in aging and stroke populations, to our knowledge, no studies have investigated this mechanism in people with PD. Investigating this mechanism in PD may therefore not only provide novel mechanistic insights for gait instability but also help refine assessment and intervention strategies targeting fall risk. Therefore, this study aims to investigate whether foot placement control is impaired during overground walking in people with PD as compared to healthy controls.

## Methods

### Dataset

This study re-analyses an existing dataset from the Laboratory for Movement Biomechanics (ETH Zurich), originally collected and presented by Mei et al. [31], who investigated the effects of electrode placement in STN-DBS on common spatio-temporal gait parameters in individuals with PD. In contrast, the present analysis focuses on the underlying control mechanisms of gait stability, specifically, the degree of foot placement control during overground walking, using a subset of the same kinematic data. Only data from participants with PD recorded prior to DBS surgery and healthy controls were included. The dataset includes 50 individuals with PD [41 male, mean age: 60 (SD 11) years, mean PD duration: 9 (5) years, mean MDS-UPDRS III in ON medication: 19 (7)] and 51 healthy controls without any known diseases or conditions [22 male, mean age: 67 (11) years] (Table 1). Inclusion criteria required participants to be between 40 and 90 years of age, free from neurological, psychiatric, and orthopaedic disorders, and able to walk independently and continuously for at least 10 min. All assessments of the PD participants were performed during ON-medication state. Participants walked barefoot continuously for 10 min at a self-selected, comfortable speed without assistance, following a path that resembled an elongated figure-8: two 10-meter segments connected by large-radius turns around two signposts. Only the straight walking segments were recorded and included in the analysis.

**Table 1:** Demographics and clinical characteristics

Characteristic	Healthy controls (*n* = 51)	People with PD (*n* = 50)
Age (years)	67 (11)	60 (11)*
Male/female	22/29	41/9*
Weight (kg)	68.1 (12.3)	76.8 (12.6)*
Height (cm)	168.9 (8.87)	176.1 (6.9)*
Disease duration	–	8.9 (4.5)
MDS-UPDRS III^a^	–	19.1 (6.9)
Levodopa equivalent daily dose (mg/day)	–	1078.8 (465.6)

Values expressed as mean (standard deviation). Differences in age, weight, and height were assessed using independent t-tests or Mann Whitney-U tests, as appropriate. The difference in the distribution of sex (male/female) was assessed using a chi-square test

Significant differences between groups are indicated by * (*p* < 0.05)

^a^Movement Disorders Society version of the Unified Parkinson’s Disease Rating Scale, section 3 (motor examination), score range 0–132, high scores indicate increased disease severity

### Data collection, processing and analysis

Kinematic data were collected at a sample rate of 100 Hz using an optical motion capture system (Vicon Nexus, version 2.3/2.8.2, Oxford Metrics, United Kingdom). A whole-body marker set consisting of 61 reflective markers was used, with at least 4 markers on each segment of the body (including feet, shanks, thighs, upper arms and forearms, pelvis, upper trunk and head) [32]. Gait events (heel-strike and toe-off) were detected using a custom algorithm based on velocity of the foot markers [33]. Consistent with previous studies of foot placement control, estimates of the CoM and foot positions were obtained using a single representative marker for each [23, 34]. The sacrum marker was used to approximate mediolateral and anteroposterior CoM position. It has been shown that this single marker can be considered a reasonable estimate of CoM position state during steady-state walking [35]. The heel marker was used to extract foot trajectories. Swing phases were identified based on the detected toe-off and heel-strike events and subsequently time-normalized to 0–100% using spline interpolation. This process was performed for both mediolateral and anteroposterior movement direction. The last step of each straight path segment was removed and an array containing all valid steps was then generated for the analysis. For each straight path segment, between 5 and 8 steps remained for the analysis.

### Foot placement model

The foot placement model, based on Wang and Srinivasan [19] and used in previous studies [21, 23, 36], was implemented using publicly available code [37]. Foot placement was characterised by step width in the mediolateral direction and step length in the anteroposterior direction. Step width was defined as the mediolateral distance between heel markers, calculated at midstance when both feet were flat on the ground. Step length was defined as the anteroposterior distance between the heel markers at the heel strike of the leading foot. Both parameters were predicted from the CoM kinematic state during the swing phase of walking using linear regression models [19]. Each time-normalized swing phase (0–100%) results in 51 equally spaced samples, representing a specific time point within the swing phase. Mediolateral and anteroposterior CoM position and velocity at each of these samples were used as predictors of foot placement. CoM displacement was calculated with respect to the stance foot, and CoM velocity as its derivative. All variables were demeaned before fitting the models for mediolateral (step width, SW) and anteroposterior direction (step length, SL):

1 $$\begin{aligned} SW & ={\beta _{po{s_{ML}}}}\left( i \right)*Co{M_{po{s_{ML}}}}\left( i \right)+{\beta _{ve{l_{ML}}}}\left( i \right)\\ & \qquad *Co{M_{ve{l_{ML}}}}\left( i \right) +{\varepsilon _{ML}}\left( i \right) \\ \end{aligned} $$

2 $$\begin{aligned} SL & ={\beta _{po{s_{AP}}}}\left( i \right)*Co{M_{po{s_{AP}}}}\left( i \right)+{\beta _{ve{l_{AP}}}}\left( i \right)\\ & \qquad *Co{M_{ve{l_{AP}}}}\left( i \right) +{\varepsilon _{AP}}\left( i \right) \\ \end{aligned} $$

The regression coefficients (β) represent the “strength” of the foot placement response to CoM state deviations from the mean. E.g. in mediolateral direction this means that more or faster lateral deviations of the CoM are followed by wider steps, whereas more or slower medial deviations of the CoM are followed by narrower steps. ε_ML_ and ε_AP_ are the residuals of the model (1 and 2) and the standard deviation of the residual is referred to as foot placement error (FPE). The FPE indicates the precision of foot placement control, i.e. how precise the feedback mechanism is executed. Relative explained variance (R^2^) of the model (1 and 2) was determined as the ratio of predicted foot placement variance and actual foot placement variance, representing the degree of foot placement control. This value reflects the proportion of foot placement that can be explained by changes in the CoM kinematic state, with higher values indicating tighter control. Both, FPE and R^2^, are measures of the quality of the control and have been shown to be sensitive to ageing and pathology [21, 23]. Each sample (i) of the swing phase represents a different normalized time point of the swing phase. Model outcomes were evaluated at two points during the swing phase: mid-swing (i = 25) and terminal swing (i = 51). Mid-swing is a point where the system can still adjust the leg’s trajectory based on sensory feedback, so it reflects feedback control. Terminal swing occurs just before the foot hits the ground (heel strike), so it reflects the final outcome of the control process, how accurately the foot is placed to maintain stability.

### Statistics

For the comparison of demographics (age, height, weight), walking speed and number of steps between people with PD and healthy older adults, either t-tests or Mann Whitney-U tests were performed, depending on the normality of the data distribution. Difference in number of male/female participants was assessed using a chi-square test. As the primary analysis, ANCOVAs were performed to test whether foot placement error and relative explained variance (dependent variables) were affected by the independent variables Group (PD vs. Control) and Timepoint (mid-swing vs. terminal swing). Since step width and step length can influence the degree of foot placement control, both parameters were included as covariates to account for their potential confounding effects [38]. Wider steps can mechanically increase stability by enlarging the base of support, whereas longer steps modify the position and velocity of the CoM relative to the stance foot. Both factors may influence the apparent coupling between CoM motion and foot placement. Including step width and step length as covariates therefore helps ensure that observed differences in foot placement control reflect genuine variations in control strategy rather than geometric or mechanical effects related to step size.

Further, in the ANCOVA it was adjusted for age effects. Gait speed strongly covaries with step length and was therefore not included as an additional covariate [39], as such collinearity can complicate the interpretation of the ANCOVA results. However, a sub-analysis including gait speed confirmed that the main group effects remained unchanged. Separate analyses were conducted for mediolateral and anteroposterior directions. Data normality was assessed using the Shapiro-Wilk test. In case of a skewed distribution, data of foot placement error were log transformed. Relative explained variance was transformed using a modified Fisher transformation. Post hoc group comparisons at each timepoint were performed using Tukey’s correction for multiple comparisons. Cohen’s d with Hedge’s correction were calculated for foot placement error to assess the effect size. As a secondary analysis, foot placement variability, i.e. how much the position of the foot varies relative to the stance foot, and CoM variability was compared between groups using Mann Whitney-U tests with Bonferroni correction for multiple comparisons. *p* values < 0.05 were considered statistically significant for all analyses.

All analyses were conducted using Matlab (version R2023b, The MathWorks Inc., Natick) and R (v4.3.1, The R Foundation for Statistical Computing, Austria).

## Results

The average gait speed was 1.20 (0.19) m/s in people with PD and 1.21 (0.14) m/s in healthy controls (*p* = 0.75). The average number of steps included in the analysis was 413 ± 123 for the PD group and 354 ± 121 for the control group (*p* < 0.005). There were significant differences (*p* < 0.05) in age, sex distribution, height, and weight between people with PD and healthy controls. Given the difference in sex distribution, a sub-analysis was conducted to examine potential sex-related effects on foot placement outcomes (Supplementary material). However, no significant differences between male and female participants were found and therefore this was not further considered in the analysis.

### Foot placement error

**Fig. 1:**
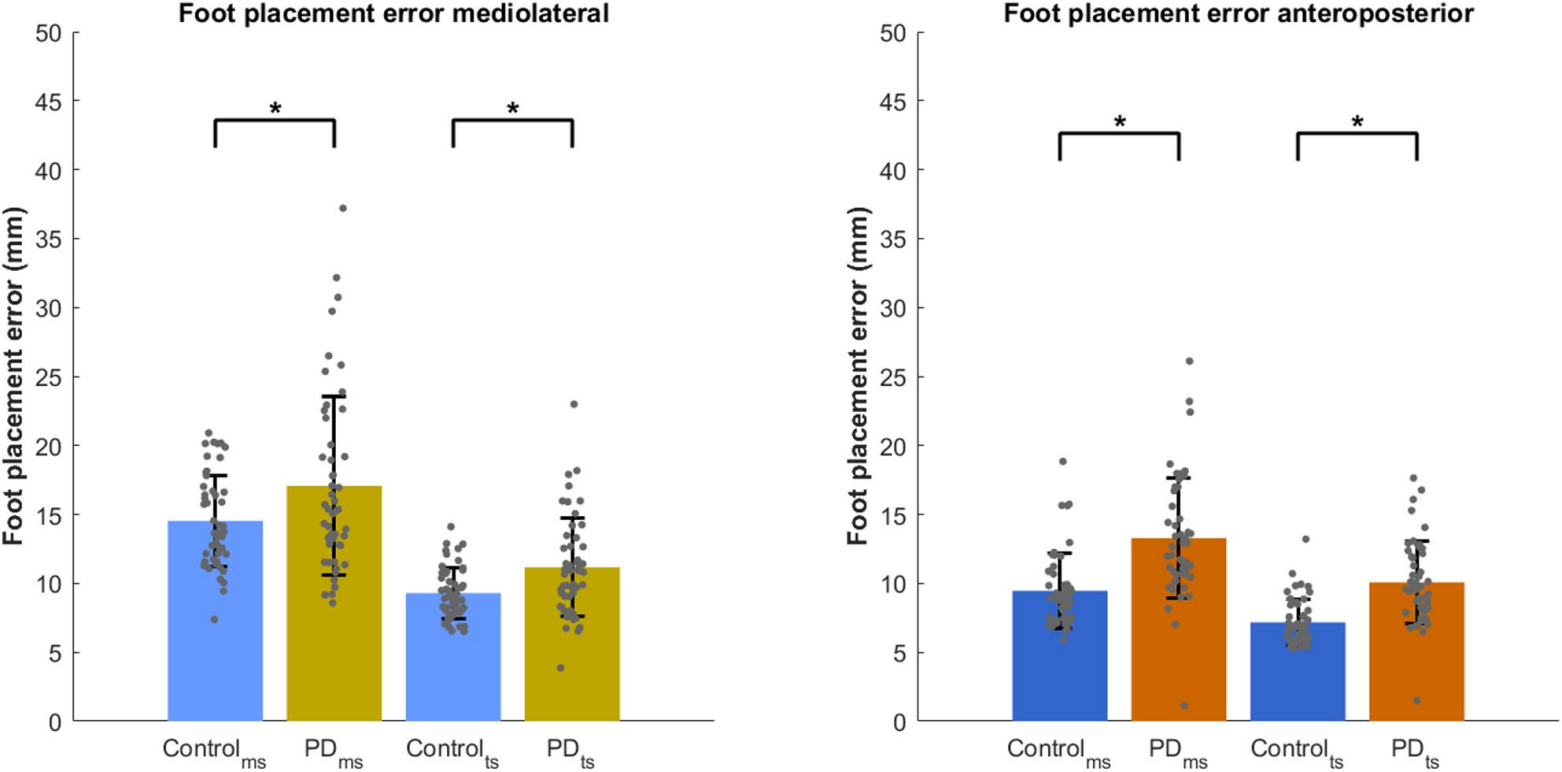
Foot placement errors (mm) for people with PD and healthy controls during mid-swing (ms) and terminal swing (ts) in both mediolateral (left) and anteroposterior (right) direction. Error bars represent standard deviation

In the mediolateral direction the overall linear model was significant (*F*(6, 195) = 24.09, *p* < 0.001). A significant main effect of both Group (PD vs. control; *F*(1, 195) = 5.35, *p* < 0.05) and Timepoint (mid-swing vs. terminal swing; *F*(1, 195) = 65.10, *p* < 0.0001) was found, while there was no significant Group × Timepoint interaction (F(1, 195) = 0.15, *p* = 0.70). This indicates that, even after adjusting for step length, step width, and age, people with PD had consistently higher foot placement errors across timepoints. Post hoc comparison confirmed that foot placement error was significantly higher in the PD group compared to controls at both mid-swing (*t*(195) = − 2.31, *p* < 0.05, Cohen’s d = 0.41) and terminal swing (*t*(195) = − 2.84, *p* = 0.005, Cohen’s d = 0.58) (Fig. 1, left). Among the covariates, longer step length was associated with higher mediolateral foot placement error (*F*(1, 195) = 10.8, *p* < 0.005).

In the anteroposterior direction (*F*(6, 195) = 15.50, *p* < 0.001), there were also significant main effects of Group (*F*(1, 195) = 20.92, *p* < 0.0001) and Timepoint (*F*(1, 195) = 17.34, *p* < 0.0001), but no significant interaction (*F*(1, 195) = 0.01, *p* = 0.94). Post hoc comparison showed that the PD group had significantly higher foot placement errors than controls at both mid-swing and terminal swing (ms: *t*(195) = − 4.57, Cohen’s d = 0.84, ts: *t*(195) = − 4.68, Cohen’s d = 1.04, *p* < 0.0001) (Fig. 1, right). Of the covariates, larger step widths were significantly associated with increased anteroposterior foot placement error (*F*(1, 195) = 12.43, *p* < 0.001).

### Relative explained variance (R^2^)

**Fig. 2:**
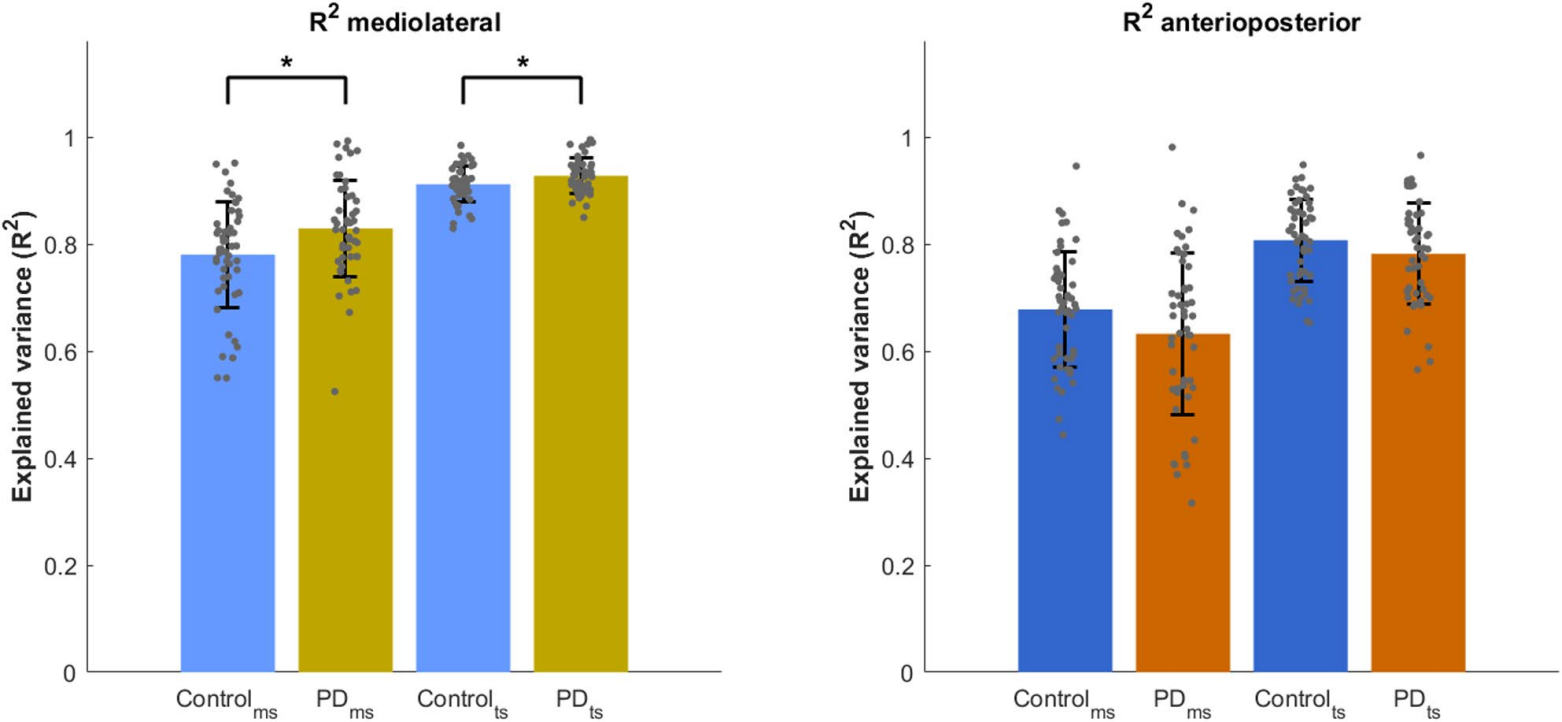
Relative explained variance (R^2^) for people with PD and healthy controls at mid-swing (ms) and terminal swing (ts) for both mediolateral (left) and anteroposterior (right) direction. Error bars represent standard deviation

In the mediolateral direction (*F*(6, 195) = 28.54, *p* < 0.001), a significant main effect of Group (*F*(1, 195) = 10.20, *p* < 0.005) and Timepoint (*F*(1, 195) = 62.85, *p* < 0.0001) was observed after adjusting for step width, step length and age. The Group × Timepoint interaction was not significant (*F*(1, 195) = 0.32, *p* = 0.57), indicating that the effect of group was consistent across both timepoints. CoM state at mid-swing explained 82% of the variance in subsequent foot placement in people with PD compared to 78% in the control group (*t*(195) = − 3.19, *p* < 0.005) (Fig. 2, left). The same held for terminal swing, where the CoM state accounted for significantly more foot placement variance in PD (93% vs. 91% in controls, *t*(195) = − 2.42, *p* < 0.05). The outcome was significantly influenced by step width (*F*(1, 195) = 41.86, *p* < 0.001), with wider steps being associated with higher R^2^.

In the anteroposterior direction (*F*(6, 195) = 12.31, *p* < 0.001), the CoM state at mid-swing explained 63% of the variance in foot placement in people with PD and 67% in controls, increasing to 78% and 80%, respectively, at terminal swing (Fig. 2, right). No main effect of Group or Group × Timepoint interaction was found, but there was a significant main effect of Timepoint (*F*(1, 195) = 31.57, *p* < 0.001), indicating that the increase in R² from mid-swing to terminal swing occurred similarly in both groups.

### Secondary outcomes

To test whether changes in R^2^ were (partially) due to increases in variability [40], differences in foot placement and CoM variability were tested, showing a significantly higher variability of step width/step length and CoM position in PD compared to controls for both ML and AP (step width/step length: *p* < 0.05, CoM position: *p* < 0.001) (Table 2).

**Table 2:** Center of mass and foot placement variability (mm) for both mediolateral and anteroposterior direction

	Healthy controls	People with PD
CoM variability ML (mm)	15.28 (3.69)	21.72 (10.55)*
CoM variability AP (mm)	13.28 (3.28)	15.55 (3.56)*
SW variability (mm)	39.90 (10.90)	49.67 (20.57)*
SL variability (mm)	20.76 (7.31)	29.61 (11.45)*

Significant differences between groups are indicated by * (*p* < 0.05)

## Discussion

This study investigated foot placement control during overground walking in people with PD compared to healthy older adults. Our results revealed that people with PD exhibit significantly higher foot placement errors in both the mediolateral and anteroposterior directions, at both mid-swing and terminal swing phases. Relative explained variance in mediolateral direction is increased compared to healthy older adults. Together, our study documents novel aspects of impaired foot placement control (feedback control) in people with PD.

People with PD had higher foot placement errors than healthy older adults. This may result from either an impairment in the integration of sensory modalities or impaired neural control of gait. An impaired integration of sensory modalities could potentially lead to an imprecise estimation of the CoM state [29]. As a result, people with PD may place their feet less precisely, limiting their ability to adequatly compensate deviations in CoM movement through appropriate foot placement. Such impaired adaptatation could contribute to reduced stability and an increased risk of falls. An impaired neural control of gait in PD can result from changes in the beta band, which play a crucial role for gait control [41, 42]. Beta oscillations in the sensorimotor cortex have long been recognized as key signatures of movement, closely linked to sensorimotor integration control [43]. For example, in healthy adults, beta activity is involved in stabilization of gait [44]. In PD, the function of beta activity is altered and influenced by levodopa, leading to an impaired neural gait control and less stable gait [45]. In addition to sensory and neural deficits, biomechanical factors may also contribute to impaired foot placement. Previous studies have shown that people with PD exhibit altered foot strike angles and reduced toe clearance at the end of the swing phase compared to healthy older adults [46]. These changes may reflect impaired motor control of the swing leg, possibly influenced by the same sensorimotor deficits, and could constrain the ability to execute precise foot placement. However, such associations remain to be investigated.

The findings of this study demonstrate a difference in foot placement error of approximately 3 to 4 mm between people with PD and healthy older adults. While the absolute difference may seem modest, it represents a substantial relative change given that typical foot placement precision ranges from 8 to 15 mm, highlighting the potential clinical relevance of even small disruptions in feedback control. Variability in step width has been shown to relate to the precision of foot placement [47]. Healthy young adults can increase kinematic variability to enhance the precision of their foot placement. A certain degree of variability is therefore beneficial for adaptability. In individuals with PD, however, whose gait variability is already increased, excessive step width variability may reduce their ability to adapt when high precision is required, thereby increasing the risk of falls [47–49]. Furthermore, Magnani et al. investigated how vestibular stimulation affects gait stability and found that even small differences in foot placement error between similar conditions were associated with reduced stability [40]. Despite the absence of a direct causal relationship being investigated, this emphasises the critical role of precise foot placement in maintaining stable gait. Therefore, the differences in foot placement precision evident in this study may bear significant clinical implications, as even modest reductions in precision might have the potential to compromise gait stability and elevate the risk of falling.

Regarding the relative explained variance in mediolateral direction, people with PD exhibited a higher degree of foot placement control (indiciated by a higher R^2^), suggesting that mediolateral foot placement in PD may be more strongly governed by CoM state than in healthy adults. This would indicate a more tightly regulated foot placement based on CoM state in the mediolateral direction among people with PD. However, R^2^ is influenced by the signal-to-noise ratio and the increased variability in foot placement and CoM position, which we observed in our data and is common in PD, can increase errors and inflate R^2^ values [40]. Therefore, the elevated explained variance in the PD group may not necessarily indicate enhanced control, but could instead reflect greater movement variability. On the other hand, there may also exist an optimal level of control, beyond which movement may be more likely to become rigid, with tighter control potentially leading to an inability to adapt to disturbances [50]. During gait, the moving base of support is narrowest in the ML direction [51]. In the single-limb stance phase, the center of mass moves closest to the lateral edge of the base of support and therefore regulation in the mediolateral direction may be challenging [20]. People with PD may compensate for this stability challenge by tighter control of ML foot placements, indicated by an increased explained variance, and decreased degrees of movement freedom. However, it remains to be investigated whether this increase is due to methodological issues arising from higher variability or indeed a higher level of control, whether beneficial or detrimental, in people with PD. No difference was found between people with PD and healthy older adults in anteroposterior direction.

Participants with PD in this study exhibited relatively mild impairments, as indicated by walking speeds that did not significantly differ from those of healthy controls. While this allows group comparisons without walking speed confounding the results [36], it also limits the generalizability of the findings to individuals with more advanced disease. However, it is expected that impairments in foot placement control would be more pronounced in people with more advanced symptoms. We investigated foot placement control during steady-state walking and it remains uncertain to what extent this control can be generalized across different walking situations, as other stability strategies may be employed during turning. Although we ensured that the overground walking steps were straight ahead (the curved parts of the figure-8 shapes were excluded), we cannot ascertain that participants did not already adjust their steps in anticipation of the turn. Curved and straight line walking differ in several aspects, such as asymmetries between the inside and outside leg, slowing down or mediolateral displacement of the CoM during turning [52]. To minimize such potential effects the last step of each straight walking path was excluded. However, given the short walking distance it was not possible to remove more than one step at the end of each path. Therefore, some included steps may not reflect steady-state walking, potentially affecting the foot placement outcomes. Only the heel markers were used to extract foot trajectories. However, it should be noted that alternative balance strategies, such as toeing-out [53], may influence foot placement control and contribute to mediolateral stability. These strategies were beyond the scope of the present analysis and should be investigated in future work.

In conclusion, our results provide evidence that feedback control is impaired in people with PD, as indicated by a less precise coordination between foot placement and CoM kinematics, which appears to be consistent across both anteroposterior and mediolateral movement directions. Given the association of foot placement control with gait stability [20], the present results may be an early step toward a mechanistic understanding of gait instability in PD, which is important for the development of effective approaches in gait rehabilitations. Designing interventions that target foot placement control and increase the precision of foot placement might improve gait stability in people with PD. Further research is needed to identify how impaired sensorimotor integration in PD affects the successful execution of this foot placement strategy.

## Supplementary Information

Below is the link to the electronic supplementary material.

Supplementary Material 1.

## Data Availability

Please contact the corresponding author for requests regarding data sharing and collaboration.

## Abbreviations

PD: Parkinson’s disease
CoM: Center of Mass
SD: Standard deviation
MDS-UPDRS: Movement Disorders Society version of the Unified Parkinson’s Disease Rating Scale
SW: Step width
SL: Step length
FPE: Foot placement error
ML: Mediolateral
AP: Anteroposterior
